# Detecting changes in the annual movements of terrestrial migratory species: using the first-passage time to document the spring migration of caribou

**DOI:** 10.1186/s40462-014-0019-0

**Published:** 2014-08-01

**Authors:** Mael Le Corre, Christian Dussault, Steeve D Côté

**Affiliations:** Caribou Ungava, Département de Biologie and Centre d’Études Nordiques, Université Laval, Québec, Québec G1V 0A6 Canada; Direction de la faune terrestre et de l’avifaune, Ministère des Forêts, de la Faune et des Parcs du Québec, 880, chemin Sainte-Foy, Québec, Québec G1S 4X4 Canada

**Keywords:** Migratory caribou, Migration, First-Passage Time, Signal segmentation process, Movements

## Abstract

**Background:**

Migratory species face numerous threats related to human encroachment and climate change. Several migratory populations are declining and individuals are losing their migratory behaviour. To understand how habitat loss or changes in the phenology of natural processes affect migrations, it is crucial to clearly identify the timing and the patterns of migration. We propose an objective method, based on the detection of changes in movement patterns, to identify departure and arrival dates of the migration. We tested the efficiency of our approach using simulated paths before applying it to spring migration of migratory caribou from the Rivière-George and Rivière-aux-Feuilles herds in northern Québec and Labrador. We applied the First-Passage Time analysis (FPT) to locations of 402 females collected between 1986 and 2012 to characterize their movements throughout the year. We then applied a signal segmentation process in order to segment the path of FPT values into homogeneous bouts to discriminate migration from seasonal range use. This segmentation process was used to detect the winter break and the calving ground use because spring migration is defined by the departure from the winter range and the arrival on the calving ground.

**Results:**

Segmentation of the simulated paths was successful in 96% of the cases, and had a high precision (96.4% of the locations assigned to the appropriate segment). Among the 813 winter breaks and 669 calving ground use expected to be detected on the FPT profiles, and assuming that individuals always reduced movements for each of the two periods, we detected 100% of the expected winter breaks and 89% of the expected calving ground use, and identified 648 complete spring migrations. Failures to segment winter breaks or calving ground use were related to individuals only slowing down or performing less pronounced pauses resulting in low mean FPT.

**Conclusion:**

We show that our approach, which relies only on the analysis of movement patterns, provides a suitable and easy-to-use tool to study species exhibiting variations in their migration patterns and seasonal range use.

**Electronic supplementary material:**

The online version of this article (doi:10.1186/s40462-014-0019-0) contains supplementary material, which is available to authorized users.

## Background

Long-distance migration is one of the most impressive large-scale processes in ecology, which allows animals to follow seasonal changes in resource availability [1,2] and reduce predation risk [3,4]. Several components of the migration can be assessed such as distance traveled by individuals, duration and timing of the migration, as well as the location of migration corridors and seasonal ranges (*e.g.* [5–8]). The timing of the migration is particularly crucial in the actual context of climate change, especially at high latitudes where changes are generally more drastic [9]. Indeed, climate change is likely to influence plant phenology [10], possibly leading to a mismatch between the timing of arrival on breeding areas and the peak in resource productivity necessary for the increased energy demand of lactation [11,12]. It is thus essential to develop standardized methods to determine the timing of migrations to assess how climate change is affecting migratory species.

The timing of migration can be assessed using the date when individuals reach particular landmarks during migration, such as certain meridians or parallels [7], or from the departure and arrival dates into seasonal ranges [13]. When seasonal ranges are well defined spatially, departure and arrival dates can easily be assessed using geographical boundaries [13,14]). Seasonal ranges, however, may vary over time. Changes in winter range locations have been observed in several migratory ungulates (*e.g.* moose, *Alces alces* [6]; sika deer, *Cervus nippon* [8]) and changes in calving ground locations may also occur (migratory caribou, *Rangifer tarandus* [15]). For migratory caribou from northern Quebec and Labrador, individuals display low fidelity to their winter home range [16], and the location of the winter range has drastically changed over the last few decades [17,18]. Similar changes for the size and location of calving ground have also been reported [15,19]. Because seasonal ranges vary geographically, the use of static landmarks is often inappropriate to assess the timing of migration. In such cases, an alternative is to investigate changes in the structure (e.g. speed, direction) of the movements [20].

During the last few decades, the technology available to follow individuals remotely has greatly improved, providing useful tools to describe animal movements. At the same time, new methods have been developed to analyze telemetry data [21] allowing for the detection of changes in the scale or pattern of movements, and linking these changes to individual characteristics or habitat heterogeneity [22,23]. One such method is the First-Passage Time (FPT [22]), which estimates the search effort of an animal along a path and discriminates between traveling and foraging activities [24]. The FPT relies on the assumption that, within a patchy environment, a consumer should concentrate its search effort in areas of interest, expressing an area-restricted search behaviour, *i.e.* slowing down and increasing its turning rate inside resource-rich patches [25]. FPT allows for the assessment of the spatial scale at which individuals select their habitat and the identification of areas where they concentrate their search effort [22,24]. At large scales, FPT can be useful to identify seasonal ranges (restricted search area) and migration routes (long-distance movements). The shift between high FPT occurring in residency areas and low FPT observed during migration can then be used to identify the departure and arrival dates of long-distance migrations [26]. Shifts in the FPT time series can be assessed visually, but Barraquand and Benhamou [27], following Lavielle [28], proposed an objective method to identify the breakpoints in the time series by combining an approach similar to FPT with a signal segmentation process.

Here we modified the approach suggested by Barraquand and Benhamou [27] by applying the signal segmentation process to FPT profiles, and propose an objective method to assess the timing of migration. Using only changes in movement patterns without the need to take into account any landmark or past seasonal ranges to define timing and patterns of migration, our approach could be useful to study the migration of species showing high variations in their migration patterns and seasonal range use (*e.g.* [29,30]). We tested the effectiveness of our approach using simulated paths with two seasonal ranges and two migrations interrupted by a stopover, to which we applied the FPT analysis and segmentation process. Then we applied the same approach to telemetry data to investigate the spring migration of migratory caribou from the Rivière-George (RGH) and the Rivière-aux-Feuilles (RFH) herds in northern Québec and Labrador. These caribou undertake a long-distance spring migration from their winter range to their calving ground with a large increase in movement rates during migration [19,31]. Our main goal was to identify dates corresponding to the departure from the winter range and the arrival on the calving ground. We first characterized the pattern of movements with the FPT method, and then used the signal segmentation process to detect segments corresponding to the winter break and the calving ground use for each individual.

## Results

### Simulations

**Figure 1:**
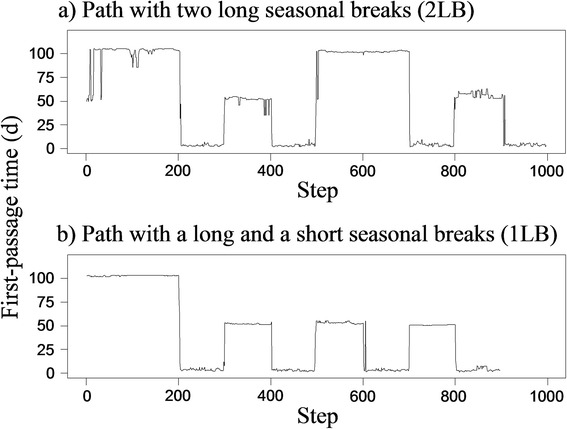
**First-Passage Time (FPT) profiles of simulated paths.** FPT is presented in relation to step number. Simulated paths are composed of 8 segments: two seasonal ranges and two migrations, each migration containing a stopover. **a)** The “2LB” paths have two long breaks corresponding to the two seasonal ranges. **b)** The “1LB” paths have one long break for the first seasonal range and one short break for the second seasonal range, similar to the stopovers.

We assessed the effectiveness of Lavielle’s method to detect changes in a FPT profile using simulated paths composed of 8 segments with two seasonal ranges and two migrations, each migration containing a stopover (1). Segmentation of the simulated paths succeeded for most of the paths when the optimal number of segments was used (Table 1). When the segmentation was constrained at 4 segments, it failed for 12% of the paths for 2LB (paths with two long seasonal breaks and two short stopovers, Figure 1a) and only succeeded for one path for 1LB (paths with a long and a short seasonal breaks and two short stopovers, Figure 1b) (Table 1). Over-segmentation occurred only on long break segments but never led to the failure of the segmentation. Precision between segmentation with the minimal length of the segments *l.min* = 1 and segmentation with *l.min* = 10 was the same when we excluded the paths for which detection failed for 2LB, and was not significantly different for 1LB (t = −1,0, df = 49, P = 0.32). Precision was higher for the unconstrained segmentation than for the segmentation constrained at 4 segments (t = 2.8, df = 41, P < 0.001).

**Table 1:** **Success and precision of the segmentation process for simulated migratory caribou paths**

**Path type and parameters used for segmentation**	**% of paths successfully segmented (** ***n*** **= 50)**	**Number of segments ± SD**	**Precision ± SD**
2LB simulations			
Min. Segment length = 1	96	8.2 ± 0.9	96.4% ± 2.2
Min. Segment length = 10	98	8.2 ± 0.8	96.4% ± 2.2
Number of segments = 4	88	4 ± 0.0	95.3% ± 2.6
1LB simulations			
Min. Segment length = 1	100	8.5 ± 0.6	97.7% ± 1.5
Min. Segment length = 10	100	8.5 ± 0.6	97.9% ± 1.1
Number of segments = 4	2	4 ± 0.0	NA

2LB simulations correspond to simulated paths with two long breaks (use of seasonal ranges) and two short breaks (stopovers). 1LB simulations correspond to paths with a long break for the first seasonal range and short breaks for the second seasonal range and the stopovers. The precision corresponds to the proportion of locations assigned to the appropriate segments for each path.

### Winter break detection

**Figure 2:**
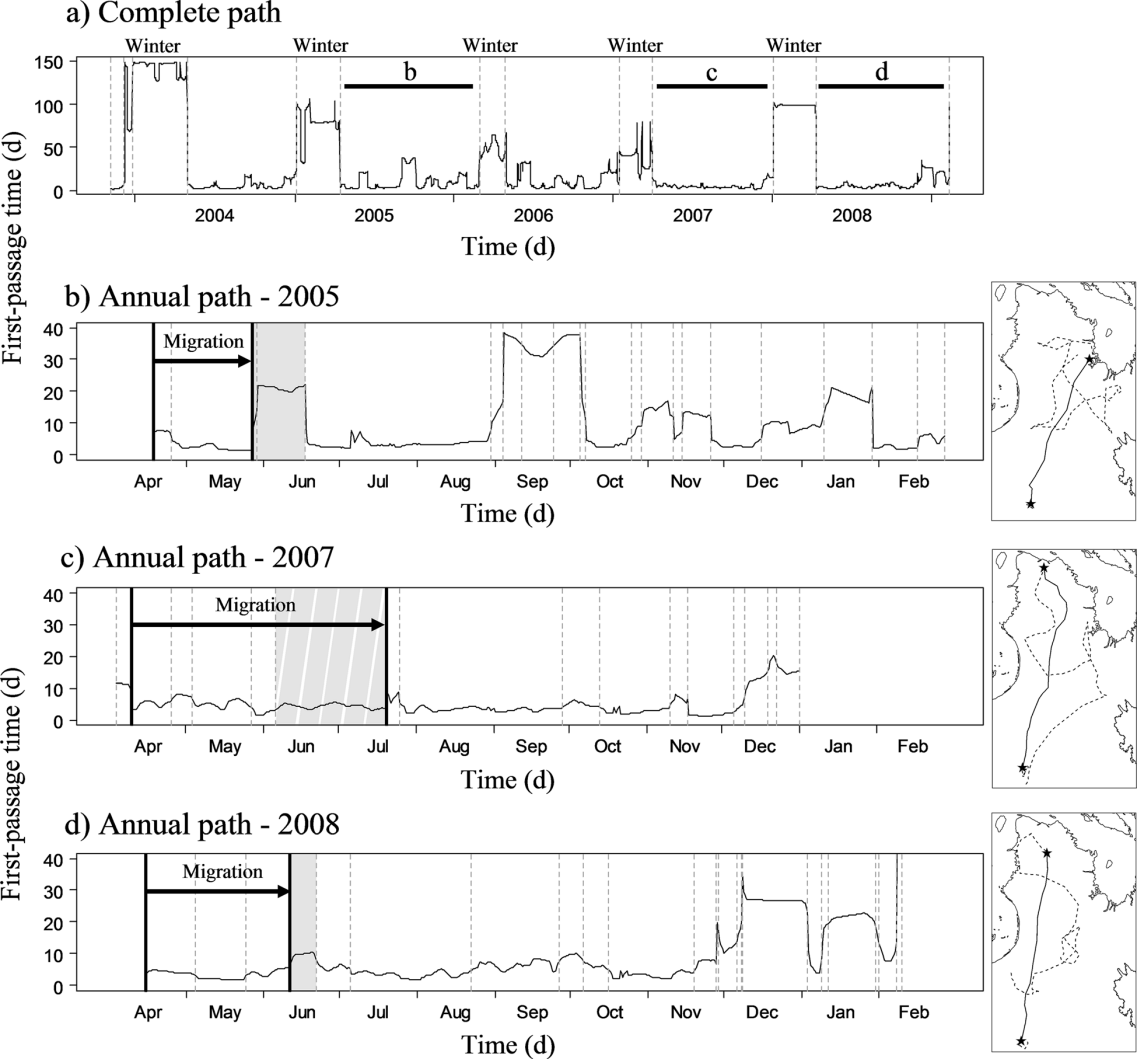
**Example of the segmentation process of a First-Passage Time (FPT) profile.** FPT profile from a female migratory caribou followed from winter 2004 to winter 2009. FPT is presented against time in days **(d)**. Dashed vertical bars represent breakpoints. **a)** First run of the segmentation: winter breaks are identified and correspond to the segments with high FPT. The segments between two winter breaks were extracted. Horizontal bars topped by a letter indicate the segments used in **b)**, **c)** and **d)** to illustrate the fine scale segmentation. **b)** Second run of the segmentation on the 2005’s path: solid vertical bars correspond to the beginning and the end of the spring migration. The migration started directly from the winter break and stopped with the beginning of the calving ground use detected in June (grey shade). The corresponding inter-winter path is represented on the map. The black line corresponds to the migration with the departure and arrival dates (black stars), and the dashed line corresponds to the rest of the inter-winter path. **c)** No calving (2007): a break is present at the beginning of the inter-winter profile, the migration starts at the end of this break. Despite a segment present in June (striped grey shade), no break was detected and the end of the migration was assessed by observing a change in path orientation. **d)** Uncertain calving ground use (2008): despite a short break was detected in June (grey shade), FPT value remained low. A visual control on the path was performed to confirm that a break occurred within the historical calving ground.

The first coarse segmentation allowed us to highlight path segments corresponding to the winter breaks (Figure 2a). We identified 679 winter breaks among 773 potential winter breaks during the first phase of the segmentation analysis. From these breaks, 538 included clear starting and ending locations, and could be considered as complete. For 141 breaks either the beginning or the ending portion of the path was missing, making the break period incomplete. The 94 remaining winter breaks (77 complete, 17 incomplete) were all detected by segmenting the yearly paths. Winter breaks were more difficult to detect in years when animals kept moving at medium movement rates, performing less marked winter breaks. Indeed, breaks that we could not detect in the first run of the segmentation were shorter (detection succeeded: 109 ± 1 days (mean ± SE), detection failed: 77 ± 5 days; Additional file 1, Additional file 2a) and had lower FPT values (detection succeeded: 54 ± 1 days, detection failed: 21 ± 1 days; Additional file 1, Additional file 2a) compared to detected breaks. Portions of the path included between two consecutive winter breaks (referred as “inter-winter path” thereafter) were extracted and segmented at a finer scale to detect calving ground use.

### Departure and arrival dates of the spring migration

We considered the departure from the winter area as the beginning of the spring migration, *i.e.* the breakpoint corresponding to the end of the winter break, and the arrival on the calving ground, *i.e.* the breakpoint corresponding to the beginning of calving ground use, as the end of the migration. Migratory movements mostly start directly from the winter break (Figure 2b and d), but in some years short pre-migration movements could occur. In these cases, we observed a segment with higher FPT value than for the migration segment at the beginning of the inter-winter path and we considered the migration started at the end of this break (Figure 2c). For the arrival date, we identified the break occurring in June that we assumed to correspond to arrival on the calving ground.

We directly identified calving ground use and late breaks on FPT profiles in 593 cases (584 complete, 9 incomplete) among 669 potential breaks corresponding to calving ground use. We did not detect any break for 5 inter-winter paths (0.7% of all inter-winter paths) where caribou did not perform a spring migration and for 10 inter-winter paths (1.5%) for which females did not significantly reduce movement rate (Figure 2c). However in 8 cases, females reversed their movements at the end of the migration. For the 61 remaining potential breaks (9%), detection failed and additional visual examination was required. Similar to winter breaks, the duration of these breaks was shorter (P < 0.001; detection succeeded: 28.3 ± 0.5 days, detection failed: 20.0 ± 2 days; Additional file 1, Additional file 2b) and the mean FPT lower (P < 0.001; detection succeeded: 20.5 ± 0.3 days, detection failed: 13 ± 1 days; Additional file 1, Additional file 2b) than for the breaks directly identified on the FPT profiles.

Overall, we identified 672 spring migration departure dates from the 773 winter breaks detected (RGH: 369, RFH: 303). The end of the break was missing in 13% of the cases, and the arrival dates were identified for all of the 593 calving ground use detected (RGH: 330, RFH: 263). We identified both departure and arrival dates for 89% of the 625 complete spring migrations present in our database (RGH: 305, RFH: 249).

## Discussion

Migratory species are currently of central interest (e.g. [9,32]), and it is thus essential to develop standardized methods to characterize migration patterns. Our approach adapted from Barraquand and Benhamou [27], based on changes in movement patterns, allows for the objective segmentation of animal paths into homogeneous bouts in order to determine the timing of the spring migration at the individual level. By detecting winter breaks and calving ground use along migratory caribou paths, we were able to identify the departure and arrival dates of the migration for most individuals.

Segmentation of the simulated paths was highly efficient without constraining the number of segments. We did not notice any difference in precision when setting a minimum segment length, however using a minimum segment length could limit over-segmentation such as observed in Figure 2b. When we used a priori knowledge of two seasonal ranges and two migrations to fix the segmentation, results were less conclusive. The success and precision of the segmentation were acceptable for 2LB as the seasonal breaks corresponded to the main breaks. However, we missed the information about stopovers. The complete failure of the segmentation for 1LB when the number of segments was set at 4, was obviously due to the fact that the second seasonal break did not differ from stopovers. We found that using *a priori* knowledge can lead to a wrong segmentation if unexpected breaks occur in the FPT profile. *A priori* knowledge of the ecology of the species should be used to interpret the segmentation. Knowing when animals are supposed to stop or to move allows identifying which segments correspond to seasonal range use and migration, and unexpected breaks or fast movements could highlight unknown behaviour or revealed disruptions of the migration or perturbations on the seasonal range.

Segmentation of the caribou FPT profiles yielded results consistent with the literature on spring migration, as well as the use of winter range and calving ground [19,31]. Individual movement rates are known to decrease below 5 km per day during two periods of the year, first on winter ranges where they are the lowest observed throughout the year [31] and second, for a short period after calving [19]. The segmentation process highlighted path segments with very high FPT values in winter and shorter pauses in June. We detected 100% of the expected winter breaks, including the breaks detected within the yearly subsets when the whole paths segmentation failed, and 89% of the expected calving ground use. Most failures at detecting winter breaks were related to individuals performing short stops with a low mean FPT. For calving ground use, failure seemed to correspond to individuals that only slowed down without stopping. Because the FPT value is lower for calving ground use than for winter breaks we used a two-step segmentation approach. The contrast between winter breaks and the other annual periods was too high to allow the segmentation to correctly detect winter breaks and calving ground use on the same FPT profile. For species showing similar space use throughout the year, with consequently similar FPT values on winter and summer ranges, a single segmentation process of the FPT profile could be sufficient. However, if space-use patterns of the seasonal ranges vary greatly, we suggest performing first a large-scale segmentation to identify and exclude the main breaks before applying the segmentation to the rest of the year.

FPT analysis has mainly been used to study foraging behaviour in marine mammals and sea birds [24,33] and, to a lesser extent, terrestrial mammals [23,34]. However, the FPT was recently used in the study of long-distance movements such as migration [26]. In these studies, circle size to calculate the FPT was generally assessed for each individual but a common scale can also be used for all individuals [35,36]. We used the same circle size, based on the peak in mean variance of FPT for all individuals [36], to facilitate the comparison between individuals. The circle size used was half the size of the mean winter range (major axis of winter home ranges: 100 ± 3 km) but it was similar to the size of the calving ground (major axis of calving ground: 55 ± 1 km). The use of an overly large radius could favour the incorporation of high-speed movement steps, resulting in smoothing the increase in FPT and leading to an overestimation of the duration of the breaks. However, step length during migration was, on average, higher than the diameter of the circle we used (spring migration step length: 65 ± 37 km), so a circle centered on the first location of the calving ground was unlikely to include the entire migration step.

The segmentation process failed for some inter-winter paths. Failures, however, can have a biological meaning. We assumed that females end their spring migration with calving but pregnancy rates reported in these two herds in the past few decades have been lower than our detection success of the calving ground use (<80%, [37]). Thus, failures could correspond to non-gravid females that have not stopped to calve but only slow down with the herd. Failures can also highlight individuals that do not adopt a “classical” migratory behaviour and the study of these individuals could reveal alternative tactics of long-distance movements. In studies including males and females, differences in timing and distance travelled have been reported between sexes [6,38] and in ecosystems with poorly predictable resources, such as rainfall-driven ecosystems, migratory individuals can adopt long-distance movements relying mostly on nomadism instead of strict migratory movements depending on predictable changes in resource availability [39]. Variations in the segmentation of the FPT profile between individuals or between years could also reveal changes in migratory behaviour.

Our approach worked well for the spring migration as it is clearly defined by the winter break and the calving ground use. The fall migration could be more difficult to define because caribou range over larger areas than in spring (e.g. [40,41]), the fall migration spans over a longer time period than spring migration and it may be separated into several bouts [40,41]. To extend our approach to the fall migration, the first step should be to identify recurrent changes in movement patterns among individuals and years. For example, in several herds movement rates are very high in summer and drastically decline at the end of the summer before caribou begin their fall migration [42,43]. This movement pattern results in a break at the end of the summer (see Figure 2b). Thus, based on movement patterns our approach is appropriate to study labile migrations with unfixed departure and arrival areas. Yearly variations in the location of seasonal home ranges are observed in several migratory ungulates [6,44,45], notably in highly mobile species tracking changes in resources during migration and throughout a broad seasonal range [29,30]. Because it does not rely on the determination of the seasonal range location to assess timing of movements, our approach could also possibly be applied to seasonal movements of nomadic species, that are far less understood than migrant species and for which seasonal ranges do not show regular temporal and spatial patterns [46,47].

## Conclusions

Here, we proposed an objective and easy-to-use approach to identify the migratory movements of individuals. We could easily determine the main characteristics of migration (timing, duration, spatial patterns) using the segmentation technique, making our approach suitable to analyse migration patterns. Moreover, studying changes in FPT values during migration could also provide information on stopover sites, a key component for the migration of numerous species [48,49], or potential migration disruptions due, for example, to human disturbances [50]. An in-depth understanding of migration is crucial to estimate the impact of the threats migratory species are facing, as several populations have already declined [32,51] or have lost their migratory behaviour [52]. The approach we developed is a helpful tool in the challenging process of acquiring the in-depth understanding of migration patterns necessary to succeed in the conservation of migratory species.

## Methods

### Caribou herds

**Figure 3:**
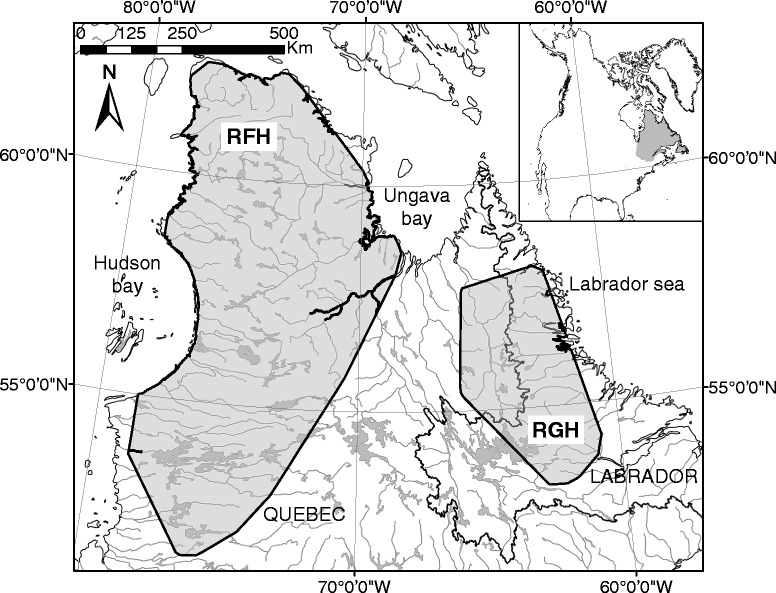
**Annual ranges of the Rivière-George (RGH) and the Rivière-aux-Feuilles (RFH) migratory caribou herds.** RGH and RFH are located in Northern Québec and Labrador. Annual ranges are 100% Minimum Convex Polygons based on ARGOS locations of females in 2010.

The RGH and RFH range over 1,000,000 km^2^ in northern Québec and Labrador (Figure 3). Although the wintering areas of the two herds may overlap in certain years, the calving grounds are located about 800 km apart (57°N, 65°W for RGH; 58°N, 73°W for RFH). Females are highly philopatric to their calving ground [53]. They leave their winter range in the boreal forest usually in April and migrate toward their respective calving ground in the tundra. Females usually arrive on the calving grounds in late May then move toward their summer range, also located in the tundra, in early July [15]. They migrate back to their wintering areas in October-December.

During the last few decades, the two herds have shown large variations in population size. RGH numbered at least 60,000 individuals in the 1950’s [54], increased up to 823,000 individuals in 1993, and then dramatically decreased to 385,000 caribou in 2001 [40] and 27,600 caribou in 2012 (Ministère des Forêts, de la Faune et des Parcs du Québec [MFFP], unpubl. data). RFH was discovered in 1975 when its size was estimated at 56,000 individuals [55]. The herd reached 628,000 caribou in 2001 [40] and declined to 430,000 individuals in 2011 (MFFP, unpubl. data).

### Tracking data

We used the locations of 252 females for RGH equipped with ARGOS satellite-tracking collars (Telonics, ARGOS platform, Mesa, Arizona, USA) between 1986 and 2012, and locations of 150 females for RFH collared between 1991 and 2012. We captured females mostly on their calving ground using a net-gun fired from a helicopter [56] following the guidelines from the Canadian Council on Animal Care. We considered individuals to be independent because capture sites within a given year were spread over several thousands km^2^. On average, we followed 44 females (SE ± 5) each year and females were monitored on average for 2.0 years (SE ± 0.1) with some individuals followed for up to 10 years. Locations were usually collected every 5 days (65.7% of the database) but frequency ranged from one location every day (1.3%) up to one per 7 days (0.9%). We filtered the data using a similar algorithm as Austin et al. [57] to eliminate aberrant locations: we selected the most accurate location for a given transmission period based on signal quality and we excluded locations leading to movements higher than 50 kilometers per day [53].

### The First-Passage Time analysis

The first step of the process was to characterize caribou movements throughout the year. For this, despite we followed Barraquand and Benhamou [27] for the segmentation process, we used the FPT analysis [22] rather than their method of residence time, derived from FPT. Both methods require setting the size of a circle used in the analysis. This circle size can be set according the ecological knowledge of the species [27] but when this knowledge is lacking, such as in our study, Fauchald and Tveraa [22] provide the methodology to assess empirically the circle size to use for the FTP analysis directly from the data set. Analysis was performed using the software R (version 3.0.0 [58]). The FPT corresponds to the time needed by an individual to cross for the first time a circle of a given radius, the individual passing by the centre of the circle. FPT values summarize both the velocity and the tortuosity of the movement along the path [22]. We associated low FPT values to long-distance movements such as migration and high FPT values to the use of seasonal ranges. To perform the FPT analysis and also the following segmentation process, we assumed that caribou moved linearly with a constant speed between two locations and completed inter-location paths by adding one point every 12 hours [22,24].

**Figure 4:**
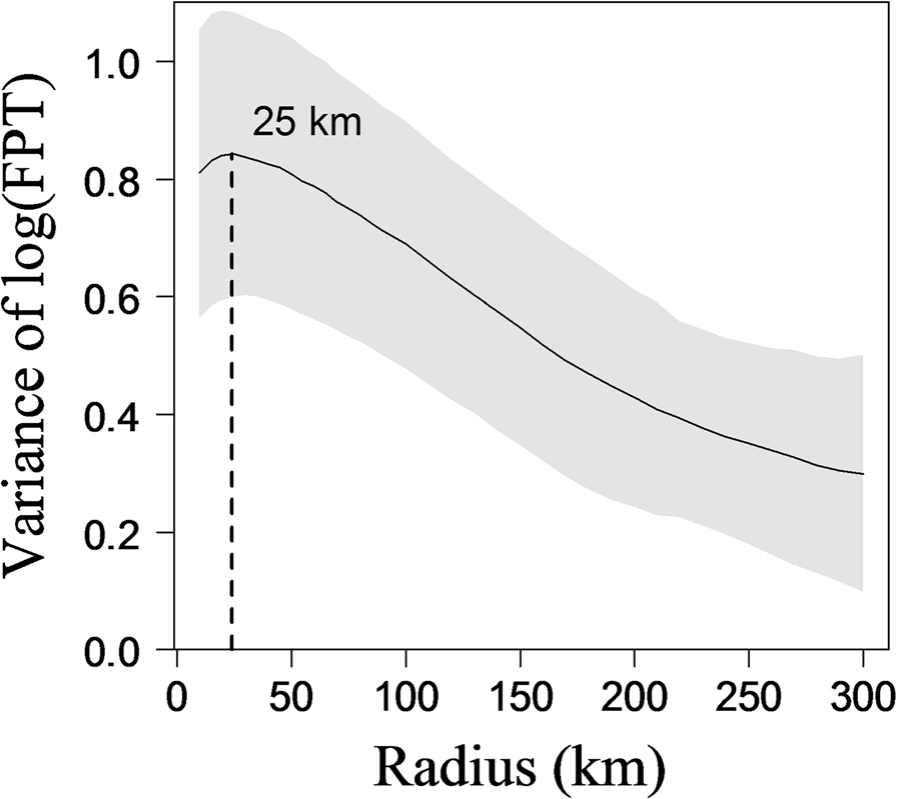
**Mean variance of the log-transformed First-Passage Time (FPT) as a function of the radius** ***r*** **.** Mean variance in log(FPT) is presented with ± SE (grey shade) and *r* is in kilometres (km). Mean variance in log(FPT) was calculated from 401 complete annual paths of migratory caribou spanning from 1 August to 31 July. The peak in variance was observed at *r*_*max*_ *=* 25 km.

Before applying the FPT to the whole data set, we first defined the radius of the circle used to calculate FPT values. From all the locations of a given female caribou, we selected complete annual paths from 1 August of a given year to 31 July of the following year between 1986 and 2010 (401 paths). We calculated FPT along each path with a given radius *r* centered on animal locations or interpolated points between them. We investigated *r*-values ranging from 10 to 300 km (radius increment: 10 to 100 km, every 5 km; 100 to 300 km, every 10 km). The radius *r*_*max*_ occurs at the peak of variance in FPT and corresponds to the spatial scale at which an individual perceives its environment [22]. We thus calculated the variance in FPT, S(*r*), for each radius and each path as Var[log(fpt(*r*))], and calculated the mean S(*r*) for each radius. We then plotted mean S(*r*) against radius, and observed a peak in the variance for *r*_*max*_ = 25 km (Figure 4). We used this radius, *r*_*max*_, as a common scale to calculate FPT along the complete path of each individual [35]. We then obtained a profile of FPT for each female by plotting their FPT values against time (Figure 2).

### Segmentation process

In the second step, we subdivided the paths in bouts of similar FPT values. Following Barraquand and Benhamou [27], we segmented FPT profiles using the Lavielle’s model selection procedure [28]. This method allows for the detection of changes in a signal by locating the breakpoints in the signal without prior knowledge on the initial number of breakpoints. Thus, the signal is segmented in bouts of homogeneous mean or variance. Lavielle’s method consists in minimizing a penalized contrast function *J*(τ,*y*) + *β*pen(τ), where *J*(τ,*y*) is the contrast function that measures the fit of τ, any segmentation of the signal, with *y*, the data set, for a given number of segments *K*. The penalty term, pen(τ), is used to assess the number of breakpoints, and *β* is a penalization parameter that weights the penalty term (for details see [28]). First, the method assesses the best segmentation of the signal for any fixed *K* segments, then the most likely segment number K* is selected*.* To assess K*, Lavielle [28] proposed to use the second derivative of the contrast function by selecting the greatest value of *K* for which the second derivative of the contrast function is greater than a given threshold *S* that we set at *S* = 0.75 following Lavielle [28] and others (e.g. [27,59]). The analysis can easily be performed using Lavielle’s program in Matlab, available at http://www.math.u-psud.fr/~lavielle/programs/. Lavielle’s method has a wider range of applications than methods based on AIC or on BIC that require Gaussian and independent data [28], and it can be applied to correlated data such as animal locations [27]. The approach is more heuristic and requires the user to make decisions regarding three parameters: the variable on which to perform segmentation (mean, variance or both), the minimal length of the segments (*l.min*) and the maximum number of segment to be inferred (*K*max). As suggested by Barraquand and Benhamou [27], preliminary examination of the FPT profiles indicated that the most important variations in our system were for mean FTP values. We therefore segmented the caribou FPT profiles according to the mean of FPT values. We set *l.min* at 1 in order to allow the procedure to detect any segment regardless of its duration. We set *K*max at 30, because Lavielle [28] suggested using a value higher than expected, *i.e.* for our study 5 segments corresponding to winter range use, spring migration, calving ground use, summer range use and fall migration. For all identified breakpoints that corresponded to inter-location points, we referred to the closest “real” location to establish the beginning and ending dates of each period.

### Simulations

We simulated two types of paths with two seasonal ranges and two stopovers (Figure 1). In the first one, referred to as 2LB thereafter, the two seasonal ranges corresponded to two long breaks (LB) and the stopovers corresponded to two short breaks (SB, Figure 1a). In the second one, referred to as 1LB thereafter, the first seasonal range corresponded to a LB and the second seasonal range to a SB (Figure 1b). This second type of path is similar to caribou paths which are composed of a main break during winter and a shorter break at calving, similar to the other breaks occurring during the year (e.g. Figure 2b). We simulated each type of path 50 times.

We built simulated paths assuming extensive movements during migration segments and area-restricted search behaviour during break segments (seasonal ranges and stopovers). We used 100 locations for the migration segments, 100 locations for SB and 200 locations for LB. Location frequency was 12 h. Based on real caribou datasets, we drew a speed value for each step from a log-normal distribution with a mean of 15 km/day and 3 km/day, respectively for migration and break segments, and with a coefficient of variation of 1 for both. We drew turning angles from a wrapped Cauchy circular distribution with a mean of 0 and a concentration parameter of 0.8 and 0.1, respectively for migration and break segments. For breaks segments, we used patches with diameters of 25 km and 50 km, respectively for SB and LB. In both cases, simulated individuals were forced to stop when crossing the edge of the patch and their next step was directed toward the centre of the patch. The edge of the patch acted as a reflecting boundary until the migration started.

We applied the FPT analysis with a circle radius of 25 km and then segmented the FPT profiles (see Additional file 3 for a comparative figure of a simulated path and a path from Argos locations). The segmentation was performed on the mean of the FPT values and we set *K*max at 30. For the *l.min* parameter we tested the segmentation without constraint (*l.min* = 1) and with *l.min* = 10, corresponding to the diameter of the circle/mean step length [27]. Strong *a priori* knowledge on the ecology of a species could encourage people to use the expected number of segments rather than the optimal number provided by Lavielle’s method. We compared the segmentation obtained with *l.min* = 1, using the optimal number of segments and using 4 segments expected for two seasonal ranges and two migration movements. We considered that the segmentation failed when a segment in the FPT profile included both a migration and a break segment. We did not consider over-segmentation as a failure if the over-segmented portion of the profile corresponded to one migration or break segment. We estimated the precision of the segmentation for the paths for which the segmentation succeeded by calculating the proportion of locations assigned to the appropriate segments.

### Detection of winter breaks and calving ground use

We used the segmentation process to identify winter breaks and calving ground use. Assuming that individuals always greatly reduce their movement rate during the winter and calving periods [19,31], we expected to detect a total of 773 winter breaks and 669 calving ground uses over our study period for all years and both herds, because we followed several individuals for more than one year. Preliminary exploration of the FPT profiles revealed that FPT values during winter were higher than for other annual periods (see Results). To avoid any bias in the segmentation process due to the contrast in FPT values between winter and other annual periods, we performed a first segmentation on the complete path of each individual, allowing the detection of the winter break. We then extracted portions of the path included between two consecutive winter breaks and we ran a second analysis to segment the inter-winter path at a finer scale to detect calving ground use.

When we did not detect a winter break during the first segmentation, we subdivided the whole path in yearly paths centered on the winter breaks (from 1 August to the following 31 July) and performed the segmentation process again on a yearly basis. For the calving ground use, when an individual appeared to arrive late on the calving ground, *i.e.* a late break observed on the FTP profile at the end or after the usual calving period, or when the break corresponding to the calving ground use did not appear clearly on the FPT profile (Figure 2d), we used a geographic information system (ArcGIS v9.3) to determine if the break occurred within the historical calving ground.

### Statistical analysis

To investigate further why breaks were not detected for some paths, we compared the breaks successfully detected, *i.e.* segments with a high FPT value corresponding to the winter break or calving ground use directly identifiable on the FPT profiles, with those where the segmentation failed during the first run. For the winter breaks, failed breaks corresponded to breaks identified by running the segmentation process a second time on a yearly subset of the whole path. For the calving ground use, we used paths that presented a slight increase in FPT in June but that was not detected automatically. We delineated the “failed” break visually using the beginning of the increase and the end of the decrease in FPT value. We compared the duration of the break (in days) and the mean FPT value of the break (in days) between the successfully detected breaks and the one the detection failed using linear mixed models (“g*lmer*” function in the “*lme4*” package [60]) with female identity as a random factor and the detection status (yes/no) as an explanatory variable. Detection status variable was centred. We used a Gaussian error with a log-link function for the mean FPT of the winter break and for the duration and the mean FPT of the calving ground use to meet the normality of the residuals and homogeneity of variance assumptions. The frequency of locations did not affect the results (unpubl. data). All results are presented as mean ± SE.

## Availability of supporting data

The FPT function is available in the adehabitatLT package in the software R and Lavielle’s segmentation process script for Matlab is available at: http://www.math.u-psud.fr/~lavielle/programs/

## Additional files

Additional file 1:
**Comparison between successfully detected breaks and breaks for which the detection failed.** Estimates are from generalized linear mixed models for the duration and the mean First-Passage Time (FPT) of the winter break and calving ground use, with the detection status (yes/no) as the explanatory variable, and female’s identity as a random factor. The error distribution was Gaussian with an identity link function for the duration of the winter break and Gaussian with a log-link function for the mean FPT of the winter break and for the duration and the mean FPT of the calving ground use. Additional file 2:
**Duration and First-Passage Time (FPT) value of the breaks according to their detection success.** Comparison of breaks of migratory caribou detected directly by the segmentation of the First-Passage Time (FPT) profiles (Success, S) and those for which the detection failed (Failure, F) for a) the winter break and b) the calving ground use. For each panel the left side presents the differences in breaks duration (in days) and the right side represents the differences in mean FPT (in days) observed during the break. The centreline is the median, the box edges are the 1^st^ and 3^rd^ quartiles and the whiskers are the data points within the range quartile ± 1.5*(interquartile range). Additional file 3:
**Simulated and Argos paths with their corresponding First-Passage Time (FPT) profiles.** a) Simulated path: FPT in days (d) is presented against step number. Dashed vertical bars represent breakpoints. The coloured segments on the path correspond to the coloured segments on the FPT profile. b) Argos path: FPT is presented against time in days (d). For the Argos path, the first segment (red) was assessed by the first coarse segmentation of the complete path of the individual and the following segments were assessed by the segmentation of the inter-winter path.

## Abbreviations

FPT: First-passage time
RGH: Rivière-George herd
RFH: Rivière-aux-Feuilles herd
MFFP: Ministère des Forêts, de la Faune et des Parcs du Québec
AIC: Akaike information criterion
BIC: Bayesian information criterion
LB: Long break
SB: Short break
