# Supplementary material for: Detecting changes in the annual movements of terrestrial migratory species: using the first-passage time to document the spring migration of caribou
Source: Mov Ecol. 2014 Aug 1;2:19. doi: 10.1186/s40462-014-0019-0 (PMC4855333; doi:10.1186/s40462-014-0019-0)
Supplement: Additional file 1: — Comparison between successfully detected breaks and breaks for which the detection failed. Estimates are from generalized linear mixed models for the duration and the mean First-Passage Time (FPT) of the winter break and calving ground use, with the detection status (yes/no) as the explanatory variable, and female’s identity as a random factor. The error distribution was Gaussian with an identity link function for the duration of the winter break and Gaussian with a log-link function for the mean FPT of the winter break and for the duration and the mean FPT of the calving ground use. [file 40462_2014_19_MOESM1_ESM.pdf]

Additional File 1 - Comparison between successfully detected breaks and breaks for which the detection failed.

Response variable	Explanatory variable	Estimate	SE	*t*-value	*P*-value
Winter break					
Duration	Intercept	104.31	1.42	73.53	<0.001
	Detection	31.66	4.15	7.63	<0.001
FPT	Intercept	3.70	0.03	109.50	<0.001
	Detection	1.01	0.10	9.60	<0.001
Calving ground use					
Duration	Intercept	3.26	0.02	139.54	<0.001
	Detection	0.43	0.06	6.91	<0.001
FPT	Intercept	2.92	0.02	147.40	<0.001
	Detection	0.44	0.05	8.10	<0.001

Estimates are from generalized linear mixed models for the duration and the mean First-Passage Time (FPT) of the winter break and calving ground use, with the detection status (yes/no) as the explanatory variable, and female's identity as a random factor. The error distribution was Gaussian with an identity link function for the duration of the winter break and Gaussian with a log-link function for the mean FPT of the winter break and for the duration and the mean FPT of the calving ground use.
