# Supplementary material for: Detecting changes in the annual movements of terrestrial migratory species: using the first-passage time to document the spring migration of caribou
Source: Mov Ecol. 2014 Aug 1;2:19. doi: 10.1186/s40462-014-0019-0 (PMC4855333; doi:10.1186/s40462-014-0019-0)
Supplement: Additional file 2: — Duration and First-Passage Time (FPT) value of the breaks according to their detection success. Comparison of breaks of migratory caribou detected directly by the segmentation of the First-Passage Time (FPT) profiles (Success, S) and those for which the detection failed (Failure, F) for a) the winter break and b) the calving ground use. For each panel the left side presents the differences in breaks duration (in days) and the right side represents the differences in mean FPT (in days) observed during the break. The centreline is the median, the box edges are the 1st and 3rd quartiles and the whiskers are the data points within the range quartile ± 1.5*(interquartile range). [file 40462_2014_19_MOESM2_ESM.pdf]

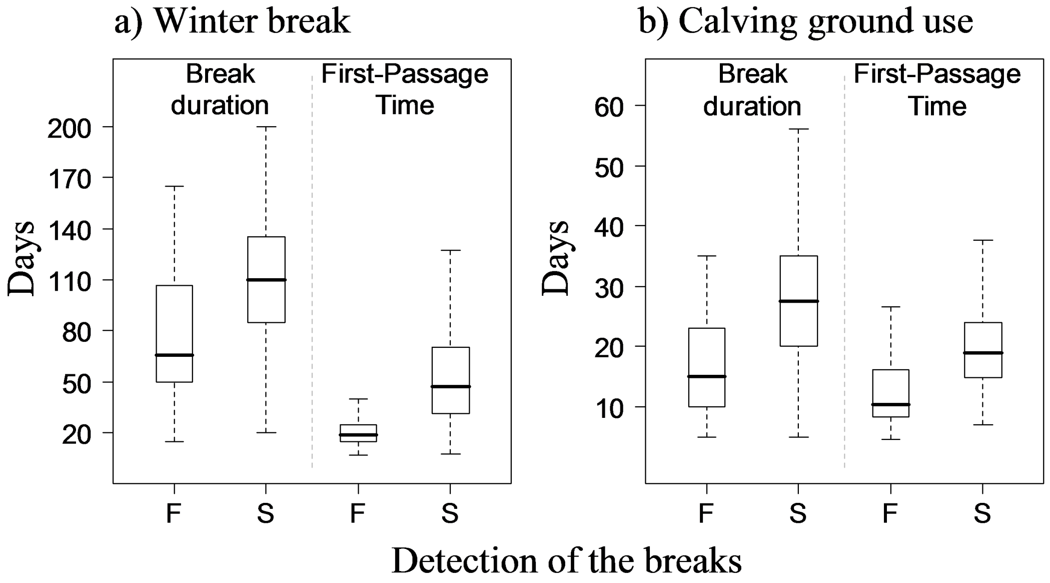

Additional File 2 - Duration and First-Passage Time (FPT) value of the breaks according their detection success. Comparison of breaks of migratory caribou detected directly by the segmentation of the First-Passage Time (FPT) profiles (Success, S) and those for which the detection failed (Failure, F) for a) the winter break and b) the calving ground use. For each panel the left side presents the differences in breaks duration (in days) and the right side represents the differences in mean FPT (in days) observed during the break. The centreline is the median, the box edges are the 1st and 3rd quartiles and the whiskers are the data points within the range quartile ± 1.5*(interquartile range).
