# Supplementary material for: Detecting changes in the annual movements of terrestrial migratory species: using the first-passage time to document the spring migration of caribou
Source: Mov Ecol. 2014 Aug 1;2:19. doi: 10.1186/s40462-014-0019-0 (PMC4855333; doi:10.1186/s40462-014-0019-0)
Supplement: Additional file 3: — Simulated and Argos paths with their corresponding First-Passage Time (FPT) profiles. a) Simulated path: FPT in days (d) is presented against step number. Dashed vertical bars represent breakpoints. The coloured segments on the path correspond to the coloured segments on the FPT profile. b) Argos path: FPT is presented against time in days (d). For the Argos path, the first segment (red) was assessed by the first coarse segmentation of the complete path of the individual and the following segments were assessed by the segmentation of the inter-winter path. [file 40462_2014_19_MOESM3_ESM.pdf]

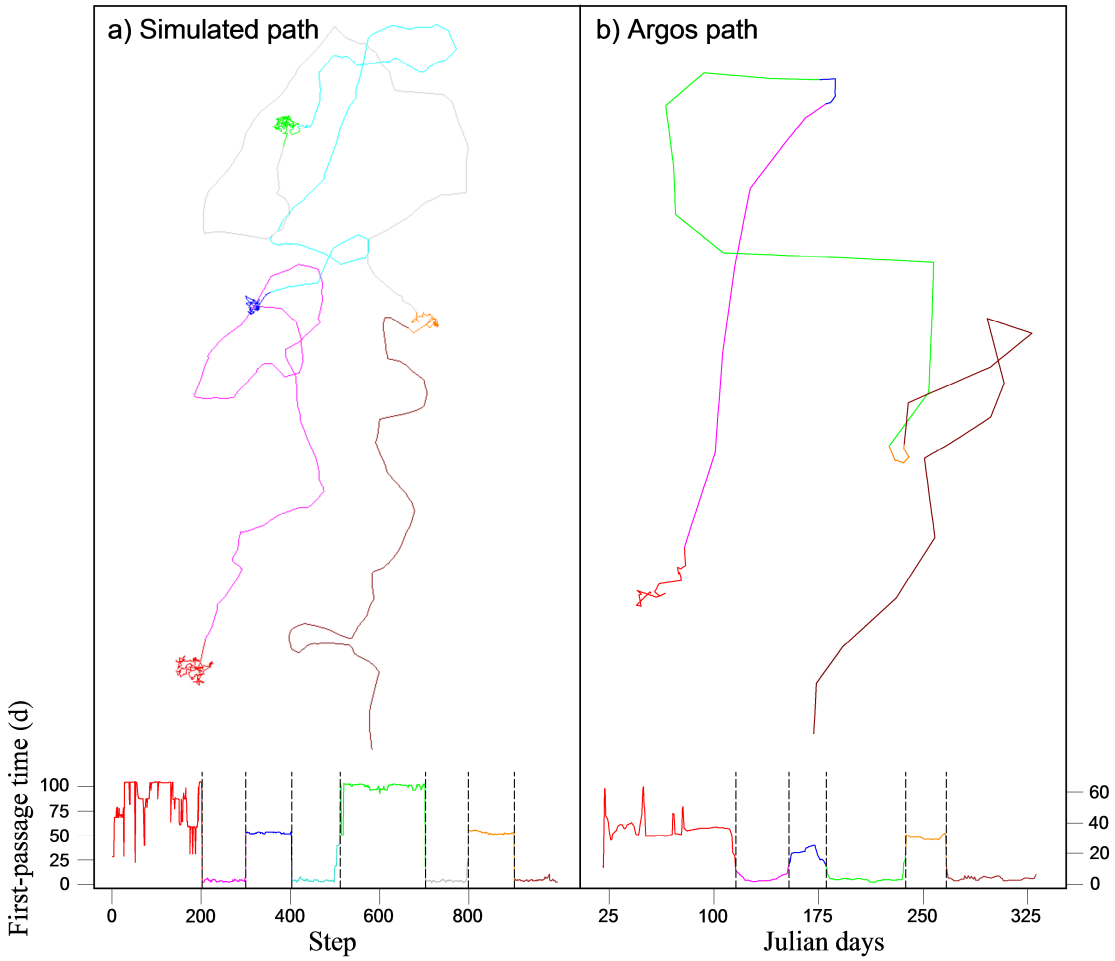

Additional File 3 - Simulated and Argos paths with their corresponding First-Passage Time (FPT) profiles. a) Simulated path: FPT in days (d) is presented against step number. Dashed vertical bars represent breakpoints. The coloured segments on the path correspond to the coloured segments on the FPT profile. b) Argos path: FPT is presented against time in days (d). For the Argos path, the first segment (red) was assessed by the first coarse segmentation of the complete path of the individual and the following segments were assessed by the segmentation of the inter-winter path.
